# Response to Berry et al’s “Cutaneous small-vessel vasculitis following single-dose Janssen Ad26.COV2.S vaccination”

**DOI:** 10.1016/j.jdcr.2021.08.041

**Published:** 2022-01-21

**Authors:** Natalie H. Matthews, Cayla M. Pichan, Alexandra C. Hristov, David M. Markovitz, Allison M. Darland

**Affiliations:** a Department of Dermatology, University of Michigan Medical School, University of Michigan, Ann Arbor, Michigan; b University of Michigan Medical School, Ann Arbor, Michigan; c Department of Pathology, Division of Infectious Diseases, University of Michigan Medical School, University of Michigan, Ann Arbor, Michigan; d Department of Internal Medicine, Division of Infectious Diseases, University of Michigan School of Medicine, University of Michigan, Ann Arbor, Michigan

**Keywords:** vaccine, small-vessel vasculitis, leukocytoclastic vasculitis, Johnson & Johnson adenovirus vector COVID-19 vaccine, SARS-CoV-2, COVID-19

*To the Editor:* On February 27, 2021, the United States Food and Drug Administration issued an Emergency Use Authorization for the Janssen adenovirus vector COVID-19 vaccine.^1^, ^2^ The most commonly reported adverse events of the Janssen vaccine include mild reactogenicity symptoms, including location injection site reactions, hypersensitivity, and systemic reactogenicity, like fatigue and headache.^2^ Prior vaccines, including adenovirus vector vaccines and influenza, have reported small- and medium-vessel vasculitis as potential adverse events.^3^, ^4^, ^5^ Most recently, Berry et al reported a case of cutaneous small-vessel vasculitis following the Janssen COVID-19 vaccine. Here, we present a similar case of cutaneous small-vessel vasculitis following vaccination with the Janssen COVID-19 vaccine.

A 64-year-old man with a previous history of deep vein thrombosis of his left lower extremity presented with a worsening rash for 4 days. His rash initially presented on his feet bilaterally as palpable purpuric papules and plaques, which spread up his legs and thighs, with the newest lesions distributed over his arms, abdomen, buttocks, and back (Fig 1). The lesions were tender to palpation and were more pronounced, edematous, and tender on the left leg compared with the right. Ten days before rash onset, the patient received his Johnson & Johnson (Janssen) COVID-19 vaccine. He denied any complications with previous vaccines, including influenza vaccines.

**Fig 1:**
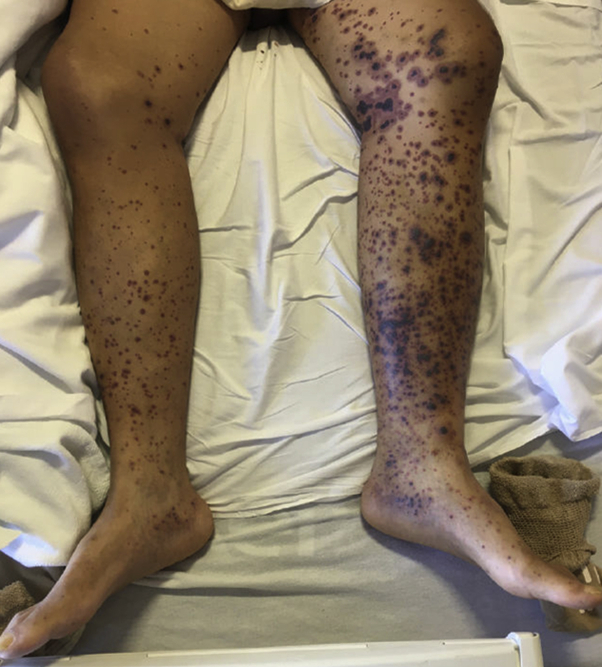
Clinical appearance of leukocytoclastic vasculitis. Bilateral legs and thighs with multiple purpuric papules and plaques, more heavily concentrated on the left lower extremity.

The patient was afebrile. His review of systems was otherwise negative. The only medication he took was warfarin, and he denied any new or changes in supplements or medications. The patient denied a history of autoimmune disease or recent infections. He was up to date on age-appropriate cancer screenings. Connective tissue and autoimmune laboratory workup were negative, including antinuclear antibody, anti-double-stranded DNA, perinuclear and cytoplasmic antineutrophil cytoplasmic antibodies, rheumatoid factor, and extractable nuclear antigen panel, and complement C3 and C4 level testing was within normal limits. Hepatitis panel and HIV antigen-antibody testing were nonreactive. Hematologic, renal, and hepatic functions were within normal limits.

Biopsy from the patient’s thigh revealed fibrinoid necrosis of vessel walls with fibrin thrombi, extravasated erythrocytes, and neutrophilic inflammatory infiltrate with leukocytoclasia (Fig 2). Direct immunofluorescence microscopy of a biopsied specimen from a new lesion on the abdomen was negative for specific immune deposits. SARS-CoV-2 in situ hybridization was negative. The diagnosis of leukocytoclastic vasculitis was rendered. Initiation of high-dose corticosteroids led to the rapid improvement of the patient’s cutaneous symptoms and clinical appearance of the rash. The patient’s vasculitis resolved and remained quiescent after the completion of his oral steroid course. His lower extremity function has returned to baseline, and there is post-inflammatory hyperpigmentation at sites of previously involved leukocytoclastic vasculitis, which are gradually improving in clinical appearance.

**Fig 2:**
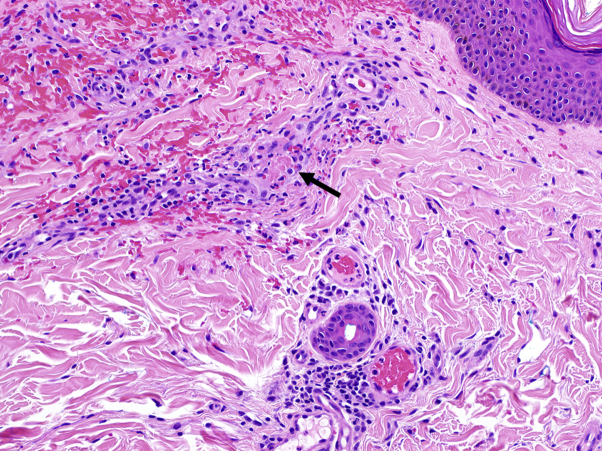
Histopathological findings of leukocytoclastic vasculitis. Vessels are surrounded by neutrophils, karyorrhectic debris, and extravasated erythrocytes and contain fibrin thrombi (A, arrow). Vessels display endothelial swelling and mural infiltration by neutrophils (hematoxylin-eosin; original magnification: ×200).

The temporal association between the patient’s vaccination and subsequent development of vasculitis in the absence of other possible inciting etiologies suggests that the vaccine may have been the trigger; however, we cannot ascribe causation. While there have been cases of systemic illness in the setting of cutaneous vasculitis,^3^^,^^4^ our patient was not acutely ill and responded rapidly to appropriate treatment.

## Conflicts of interest

None disclosed.
